# Metastasis of Rectal Adenocarcinoma to the Penis and Scrotum in an Adult

**DOI:** 10.7759/cureus.18454

**Published:** 2021-10-03

**Authors:** Thomas Schroeder, Benjamin Plambeck, Cole Bowdino, Dominick DiMaio, Andrew Christiansen

**Affiliations:** 1 Division of Urologic Surgery, University of Nebraska Medical Center, Omaha, USA; 2 Department of Pathology and Microbiology, University of Nebraska Medical Center, Omaha, USA

**Keywords:** oncology, palliative, penis, scrotum, cutaneous metastasis, rectal adenocarcinoma

## Abstract

We present a unique case of a 74-year-old male with rectal adenocarcinoma and subsequent cutaneous metastases to the penis and scrotum. Although penile and scrotal metastases have been described separately in the literature, no report has documented simultaneous metastases to both sites. Here, we describe our patient’s clinical course, treatment, and intervention throughout the timeline of his disease. We also discuss the presentation, evaluation, management, and prognosis of cutaneous metastasis to the penis and scrotum.

## Introduction

Colorectal cancer is the third most prevalent form of cancer in the United States and is projected to account for 149,500 new cancer diagnoses and 52,980 deaths in 2021 [1]. Improved screening through colonoscopy has improved overall survival and decreased the rate of metastasis at presentation [2]. When metastasis is present, colorectal cancer preferentially metastasizes to the liver and lungs before reaching other portions of the body [3,4]. 

Penile metastasis from rectal adenocarcinoma was first described by Eberth in 1870 [5] and later reviewed by Paquin and Roland in 1956 with the development of the direct venous extension hypothesis [6,7]. This remains a very rare manifestation of advanced disease, with metastasis to the penis often presenting 36 months after initial diagnosis. Scrotal metastasis from colorectal carcinoma is likewise exceptionally rare, with approximately 20 cases reported, most of which involve incisional site seeding following surgical resection [8-10]. Here, we present the case of a 74-year-old male diagnosed with rectal adenocarcinoma who developed cutaneous metastasis to both the penis and scrotum. 

## Case presentation

A 74-year-old male presented with anal pain, rectal pain, and diarrhea, which had begun two months prior to his visit. His last colonoscopy was four years prior; he otherwise had no previous surgical history. His family history was notable for a father and brother who both had suffered from prostate cancer, bladder cancer, and pancreatic cancer, both of whom were deceased. He had a 7.5 pack-year smoking history with occasional alcohol consumption. The patient consented to the use of his medical documentation for research purposes. 

He was referred to colorectal surgery where a rectal examination under anesthesia with anoscopy and biopsy revealed a 5 cm ulcerative lesion on the right anterolateral aspect of the anal canal. The lesion extended 5 cm away from the anal verge. A biopsy of the lesion showed poorly differentiated adenocarcinoma with proficient staining for mismatch repair genes (MMR) denoting a low likelihood of hereditary nonpolyposis colorectal cancer (HNPCC)/Lynch syndrome. There was also positive staining for anti-cytokeratin (CAM5.2), cytokeratin-7 (CK7), cytokeratin-20 (CK20), and caudal type homeobox transcription factor 2 (CDX2). 

The initial computed tomography (CT) imaging showed metastases to lobes 6 and 7 of the liver. Follow-up magnetic resonance imaging (MRI) showed suspicious inguinal nodes with heterogeneous but symmetric left and right inguinal lymph nodes. A 2.2 cm lesion that was suspicious for metastasis was seen in the right iliac bone. 

The patient underwent systemic chemotherapy with folinic acid, 5-fluorouracil, and oxaliplatin (FOLFOX) with bevacizumab. He also underwent cryoablation for a liver lesion. His tumor showed no growth, but after 14 months of chemotherapy with FOLFOX, the patient was found to have progression of his primary tumor upon repeat MRI scan. His chemotherapy regimen was altered, and he was started on capecitabine and external beam radiation therapy (XRT) to the pelvis, receiving a total dose of 5940 cGy over eight weeks. Following radiation therapy, the patient returned with widespread metastatic disease on imaging, involving the lungs, liver, peritoneum, and bones. His chemotherapy regimen was changed to folinic acid, 5-fluorouracil, and irinotecan (FOLFIRI) and ramucirumab for three months. His treatments were stopped for a planned diverting colostomy procedure due to severe fecal incontinence causing skin excoriation.

The patient presented to his primary care provider (PCP) three months after radiation therapy with right inguinal, scrotal, and penile cutaneous lesions along with urinary hesitancy and local irritation, swelling, and pain. The lesions were described by his PCP as vesicular, and due to concerns for *Staphylococcus aureus *and herpes zoster infections, he was treated with cephalexin and valacyclovir. The lesions persisted; were described later that month as leatherlike, thickened, and nodular; and presumed to be radiation dermatitis. 

**Figure 1:**
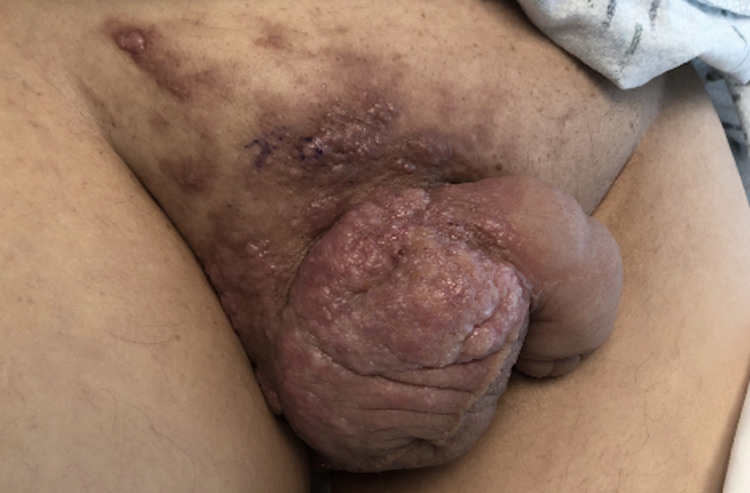
Cutaneous nodules on the hemiscrotum, penile shaft, and superior pubic area. The penis was maintained in a fixed position.

**Figure 2:**
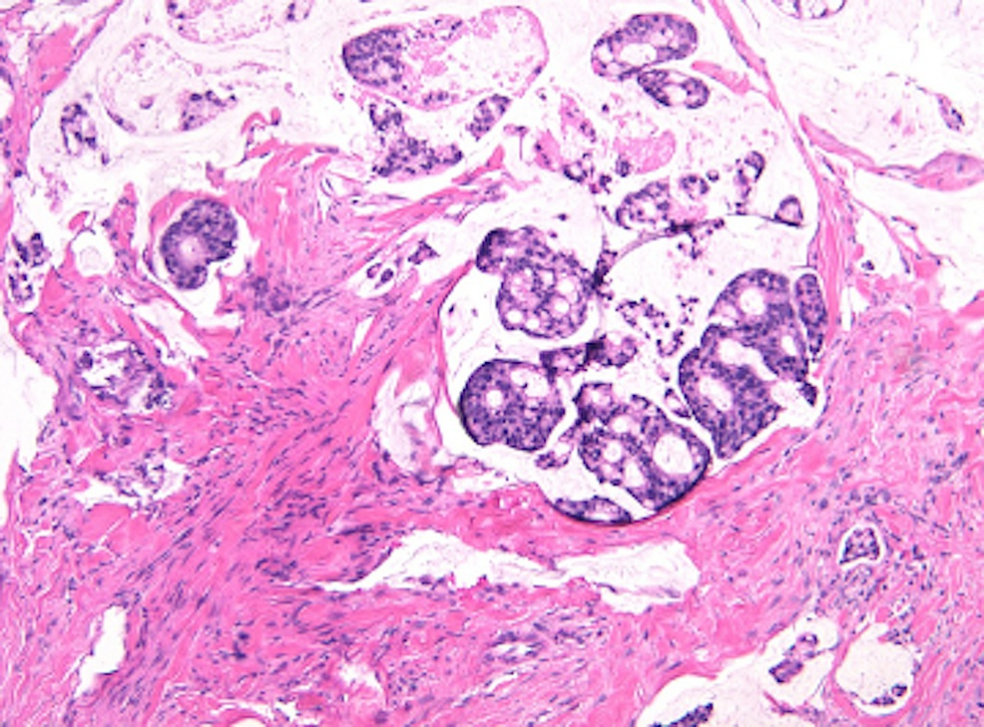
Adenocarcinoma with a cribriform architecture within pools of mucin (H&E, 100x). H&E: hematoxylin and eosin

**Figure 3:**
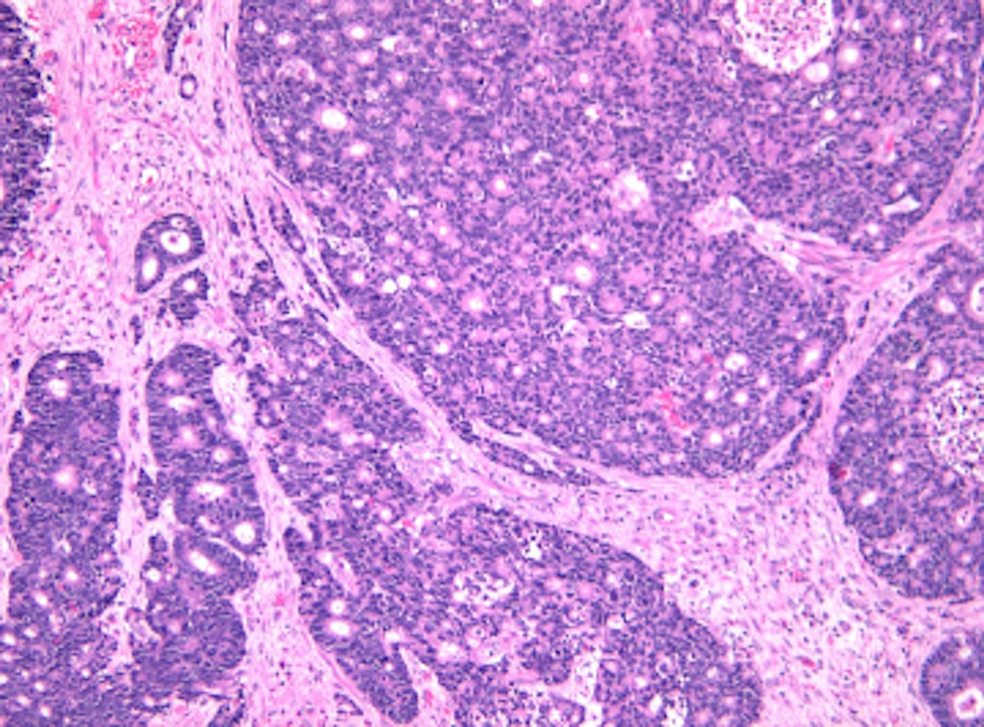
Patient’s previous anal adenocarcinoma demonstrating a similar cribriform architecture but no mucin (H&E, 100x). H&E: hematoxylin and eosin

**Figure 4:**
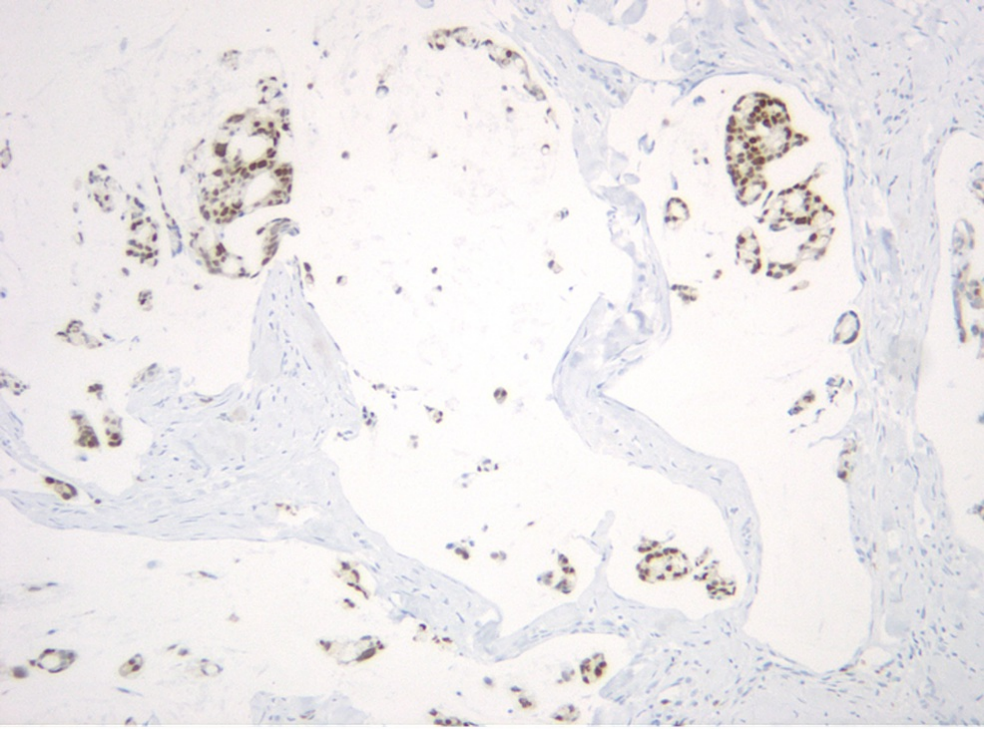
Positive staining of adenocarcinoma for CDX2, consistent with colorectal primary adenocarcinoma (CDX2, 100x). CDX2: caudal type homeobox transcription factor 2

**Figure 5:**
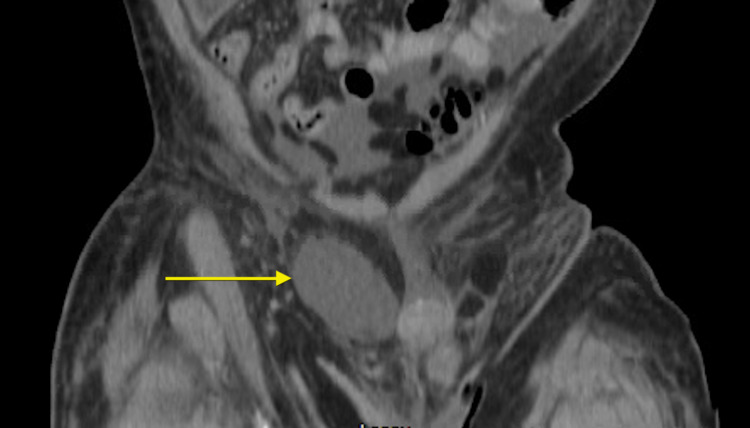
Coronal CT imaging obtained at the time of penile and scrotal metastatic presentation showing nodular mesenteric and peritoneal infiltration and thickening consistent with peritoneal carcinomatosis and a large mass present in the right hemiscrotum. CT: computed tomography

**Figure 6:**
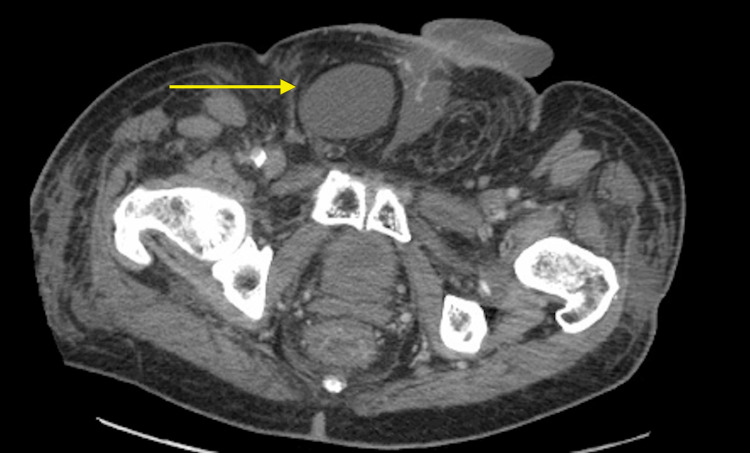
Transverse CT imaging of the patient at the time of penile and scrotal metastatic presentation displaying infiltration into the right hemiscrotum. CT: computed tomography

It was not until two months later during hospital admission for the patient’s planned surgery that dermatology was consulted to evaluate the patient’s skin lesions (Figure 1). They noted skin thickening on the penis and scrotum, and a punch biopsy of the lesion revealed adenocarcinoma and cells with a high nuclear to cytoplasmic ratio arranged in a cribriform pattern (Figure 2), similar to the previous anal biopsy (Figure 3). There was positive staining for CK7, CK20, and CDX2 (Figure 4), which was also similar to the anal biopsy. Repeat CT imaging was conducted and showed increased nodular mesenteric and peritoneal infiltration and thickening consistent with peritoneal carcinomatosis (Figures 5 and 6). The glans penis was fixed within the phimotic and edematous penile shaft skin along with an abnormal right inguinal fold, right hemiscrotum, and penile shaft. Lymphedema was present within the penile shaft and suspected to be due to local invasion of metastatic disease. Preoperative Foley placement was noted to be difficult, requiring urologic consultation, and a 14 Fr silicone catheter was placed over a guidewire due to the tortuous nature of the patient’s urethra. Following the procedure, concern for urinary retention led to the patient being discharged with a chronic indwelling Foley catheter.

Upon follow-up three weeks later with urology, his physical examination revealed an obvious gross tumor growing into the right hemiscrotum and penis. Foley catheter was continued due to continued concern for persistent urinary retention and urethral obstruction secondary to his cutaneous metastasis. Unfortunately, he declined rapidly, was placed into hospice, and passed away one week later. 

## Discussion

Penile metastasis from a primary rectal adenocarcinoma is rare, while scrotal metastasis is exceedingly rare, especially with no surgical resection of the primary tumor and the absence of incisions near the scrotum [3,4,6-11]. The combination of these two uncommon metastases is noteworthy as the glans penis, right hemiscrotum, and penile shaft were all involved in contiguous lesions in our patient. The most significant portion of the lesions involved the cutaneous tissues of the scrotum and penis. One hypothesis for the mechanism of cutaneous metastasis from rectal adenocarcinoma is seeding by retrograde venous flow due to proximal obstruction of venous structures that are shared between pelvic organs and the dorsal venous complex of the penis [3,6,11]. Other proposed mechanisms include the direct extension of the tumor along perineal planes, arterial embolization from liver or lung metastasis, or instrumentation, disrupting tumor and causing seeding [6]. Given our patient’s CT findings, the most likely mechanism was the direct extension from the primary tumor site as he had not previously received instrumentation to his genitals and the largest burden of the tumor was evident in his penis and scrotum. 

The most common symptoms of scrotal and penile metastases include malignant priapism, urinary retention, edema, cutaneous manifestations, and hematuria [11]. Our patient’s cutaneous findings and resultant lymphedema caused him to have chronic urinary retention that could only be managed with a long-term indwelling Foley catheter. The patient also experienced significant pain and swelling secondary to his lymphedema. 

The diagnostic algorithm for genital cutaneous metastases involves lesion biopsy with immunohistochemical staining and advanced imaging with CT or MRI (preferably) to assess the extent of carcinomatosis [11]. Although positive staining for CK7 is uncommon, this was present on biopsy for our patient and is consistent with metastatic mucinous colorectal adenocarcinoma. Anal biopsy from two years prior demonstrated a very similar staining pattern and appearance. CT imaging was also obtained for our patient, which showed peritoneal carcinomatosis and an obstructing mass in the right hemiscrotal cord. 

Genital cutaneous involvement is a manifestation of advanced rectal adenocarcinoma and a poor prognostic indicator, with approximately 80% of patients dying within six months, despite treatment efforts [11]. No specific management strategy has been standardized; however, potential treatments include total penectomy, surgical excision, radiotherapy, chemotherapy, and symptomatic management. From this list, the only therapy shown to have survival benefits for patients with penile metastasis is total penectomy [12]. Due to our patient’s extensive involvement of his advanced disease and excessive morbidity associated with penectomy, the urologic team decided he was not a candidate for penectomy. 

The two-month time interval from cutaneous scrotal and penile metastases to his surgical presentation requiring catheter placement caused a delay in the patient being evaluated by a urologist. At the time of his visit following long-term Foley catheter placement, concern for urinary retention and penile edema required the Foley to remain in place. Earlier recognition and urologic evaluation of this patient’s cutaneous metastases may have provided more palliative treatment strategies to improve the patient’s quality of life, such as suprapubic tube placement.

## Conclusions

Coexisting penile and scrotal metastases from a primary rectal adenocarcinoma are tremendously rare and undocumented in the literature. These entities can present with cutaneous lesions, pain, urinary retention, and edema. When cutaneous involvement is present, diagnosis typically requires lesion biopsy and advanced imaging. Although prognosis is very poor and the available treatments provide limited survival benefits, prompt diagnosis and referral for urologic evaluation is critical and could optimize palliative efforts, thus improving patient quality of life.
